# Supplementary material for: Are carbon emissions trading and green financial instruments synergistic? -Comprehensive quantitative research based on content analysis
Source: PLoS One. 2024 Mar 7;19(3):e0298601. doi: 10.1371/journal.pone.0298601 (PMC10919601; doi:10.1371/journal.pone.0298601)
Supplement: S1 Data — (ZIP) [file pone.0298601.s001.zip › Data/地级市Z_F工作报告.zip!/╔╜╬≈/┬└┴║╩╨/└┤╫╘╣1⁄2╓┌║┼í╛┬φ┐╦╩2╛▌═°í┐_2013.docx]

各位代表：

现在，我代表市人民政府向大会作工作报告，请予审议，并请市政协委员和其他列席人员提出意见。

2012年工作回顾过去一年，面对经济增长下行压力和市场持续疲软的不利形势，在市委的坚强领导下，我们深入贯彻落实科学发展观，牢牢把握“打基础、利长远、惠民生”总体要求，迎难而上，积极应对，着力推动转型跨越发展，经济社会发展和各项工作都取得了新的成绩。

1、经济保持稳定增长。全市地区生产总值完成1230.4亿元，增长10.8%，总量由全省第四位上升到第三位；工业增加值完成892.1亿元，增长13.5%，总量全省第一;固定资产投资完成690.4亿元，增长27%；全社会消费品零售总额完成301.4亿元,增长15.5%。

在宏观形势趋紧的大背景下，我市能稳中求进，企稳进位，这就有效地稳住了市域经济的基本面，并得以健康有序地发展。

2、财政收入大幅增加。全市财政总收入完成341.7亿元，增长23.4%，总量全省第二，增幅全省第一；一般预算收入完成141.9亿元，增长41.2%，总量全省第二，增幅全省第一。财政收入质量明显改善，一般预算收入占财政总收入比重达到41.6%。财政总收入超过10亿元的县又增加了1个，达到10个。财政总收入的大幅增长，使我市的经济实力大大提高，一般预算收入的大幅增长使我市可用财力增强，有力地支撑了全市各项工作的正常运行。

3、项目建设扎实推进。全市全年上马重点项目473个，总投资5456亿元，当年完成投资1550亿元，其中市里主抓的“百项重点工程”完成投资972.7亿元，全市重点工程完成额居全省第二。一些大项目基本建成或投产，兴县西山1500万吨煤矿建成投产，兴县华电1000万吨、临县霍电1000万吨、文水金地300万吨大矿全面建成，柳林凌志960万吨洗煤建成投产，孝义金州、鹏飞焦化一期建成。中钢1780m3高炉、文水海威1380m3高炉建成，岚县太钢750万吨精矿粉、200万吨球团建成投产，交口信发240万吨氧化铝建成投产，兴县中铝、孝义信发两个项目建设顺利。柳林联盛煤矸石电厂建成，交城国锦电厂抓紧建设；汾阳汾酒城建成并部分投产。庞泉沟果老峰水上乐园建成。特别是我市与国防科技大学合作的军民融合协同创新研究院正式成立，柳林李家湾光电子园、离石无人机等高科技产业项目紧张建设。省委、省政府观摩团到我市观摩项目后，给予了高度评价。

4、强农惠民取得实效。全市全年“三农”投入112.1亿元，同比增长29.5％。粮食生产实现“八连增”，总产达到11.1亿公斤。农业“六大工程”成效显著，新发展设施蔬菜2.22万亩；建成千井富民工程200眼；完成造林56.11万亩，新植核桃林51.8万亩；开工2.28万人易地扶贫搬迁工程；孝义大象集团1亿只肉鸡屠宰加工及60万吨饲料生产项目建成投产，离石、临县大象两个4500万只肉鸡养殖项目正式启动；完成350个重点村、12个集中连片示范区建设和100个贫困村整村推进任务，减少贫困人口11.1万人。省“五个全覆盖”超额完成。我市“方便农民五件实事”年度任务超额完成，共投资3.13亿元，建成幼儿园540所、澡堂706个、理发室671个、磨面房724个、红白理事厅746个，安装太阳能路灯868个村，有的县三年任务一年完，使农民的生活条件得到较大改善。参加农村养老保险、城镇养老保险、基本医疗保险达到249.6万人，参保人数均超额完成年度任务。农村低保提标扩面，每人每月提高22元，全市农村低保人数达到30万人。农民人均纯收入达到5346元，增长13.1%；城镇居民人均可支配收入突破2万元，增长14.8%。新增城镇就业5.6万人。为行政事业单位干部职工提高津贴补贴标准，使其工资水平与省直机关和太原等地拉平。由市财政出资，让47户市属国有特困企业2992名职工和1557名退休人员全部参加了城镇职工基本医疗保险。开工建设保障性住房13185套，超额完成省下达任务。投入抗灾救灾资金2.33亿元，发放救灾衣物17.23万件，有力保证了临县灾区群众的正常生产生活。节能减排任务如期完成，万元生产总值综合能耗、工业用水量、主要污染物排放量等约束性指标均控制在计划之内。全市二级以上天数达到4596天，同比增加21天，空气质量优良率达到96.6%。

吕梁城区煤气置换天然气已完成40%，有10个县（市、区）城区居民用上了清洁天然气。

5、基础设施加速建设。吕梁新城正式开工建设，我们用一年的时间进行了认真细致的规划和设计，不仅做了大量的比对和评选，还向市委常委会、市人大常委会、市政协常委会作了专题汇报，并广泛征求了广大市民的意见。目前，新城所有的规划、设计已基本完成，方山、离石已开始拆迁。吕梁民用机场即将建成。西纵高速、环城高速基本建成。农村街巷硬化完成7505公里，超出省下达任务3097公里。中南出海大通道、太兴铁路、吕临铁路等建设步伐加快。柏叶口水库建成蓄水，龙门供水按期推进，中部引黄、沿黄提灌等骨干水利工程加快建设。一批输变电和电网项目相继建成并投入运行。

6、安全稳定持续好转。深入开展以煤矿为重点的各行业各领域安全生产专项整治行动，实现了安全生产事故起数、较大事故起数、主要相对指标“三下降”，杜绝了重特大事故的发生。食品安全保障水平进一步提高。深入开展领导干部大接访、矛盾纠纷大排查、信访问题大化解“三大活动”，共排查矛盾纠纷和群众诉求55.54万件，化解55.52万件。加强公共安全管理，健全社会治安防控体系，依法打击违法犯罪，妥善应对和有力处置了各类突发事件。

7、社会事业全面进步。坚持教育优先发展，学前教育三年毛入园率达到65%，义务教育巩固提高，高中阶段毛入学率达到92.5%，中职教育免学费入学实现全覆盖。吕梁学院在校生达到18900人,排全省高校第6位。汾阳医学院护理学成为全国特色专业、省级重点学科和品牌专业。医药卫生体制改革扎实推进，基层基本药物制度实现常态化，1177个基层医疗卫生机构全部参加网上采购，县级公立医院改革试点取得实质性进展，新型农村合作医疗参合率提高到97.79%。

文化事业繁荣发展，一批文艺作品获得全国大奖，市直经营性文化事业单位及文艺院团转企改制全面完成。组织实施科技计划项目80个，消化吸收科技成果130个，争取省以上各类科技计划57项，申报专利1202项。

8、民主法制得到加强。完善重大决策规则和程序，自觉接受人大和政协的监督，积极支持各民主党派、工商联、无党派人士参政议政，共办理人大代表建议194件、政协委员提案489件。“六五”普法成绩显著，“法治吕梁”建设深入推进。强化行政监察和审计监督，加强政府系统廉政建设，深入开展煤焦、工程建设等重点领域专项治理。建成公共资源交易中心并试运行。清理取消行政审批事项300余项，查处各类违法违纪案件223件。

各位代表，取得以上成绩，是省委、省政府坚强领导、亲切关怀的结果，是市委统揽全局、科学决策的结果，是市人大、市政协大力支持、有力监督的结果，是全市广大干部群众同心同德、团结奋斗的结果，是社会各界鼎力协作、共同奉献的结果。在此，我代表市人民政府，向全市人民，向驻吕解放军指战员、武警官兵和公安民警，向各民主党派、人民团体以及所有关心支持吕梁发展的朋友，向尽心履职的各位人大代表和政协委员，表示崇高的敬意和衷心的感谢！

在总结经验和成绩的同时，我们清醒地认识到问题和差距仍然不少。一是发展中不平衡、不协调、不可持续的问题依然突出，有一些问题还没有得到有效解决。二是有的经济指标没有全面完成年初确定的目标，有一些工作落实得还不够扎实。三是综改试验没能在政策层面上取得实质性突破，还有一些不敢先行先试的顾虑。四是安全事故仍有发生，有一些监管措施还没有全部落实到位。五是一些地方和部门作风不实、效率不高，还存在一些吃拿卡要甚至违法乱纪的现象。以上这些问题，我们要引起高度重视，并加以解决。

2013年工作安排

2013年是全面贯彻落实党的十八大精神的开局之年，是实施“十二五”规划承前启后的关键一年，也是本届政府任期的最后一年。做好今年的工作，意义十分重大。

今年工作的指导思想是：以邓小平理论、“三个代表”重要思想、科学发展观为指导，全面贯彻落实党的十八大精神和全国“两会”精神，牢牢把握“打基础、利长远、惠民生”的总体要求，大力推进“四化”协调发展，突出抓好资源转化和扶贫攻坚，着力保持经济持续健康发展，着力加快产业转型升级，着力推进城乡发展一体化，着力保障和改善民生，为加快稳定脱贫、全面建成小康社会努力奋斗。

经济社会发展的主要预期指标是：地区生产总值增长11％；财政总收入增长17%；规模以上工业增加值增长13%；一般预算收入增长19％；固定资产投资增长26％；社会消费品零售总额增长16％；服务业增加值增长10%；城镇居民人均可支配收入增长15%；农民人均纯收入增长18％；居民消费价格总水平涨幅控制在3.5%左右。约束性指标是：万元生产总值综合能耗下降3.5%，万元生产总值二氧化碳排放量下降3.7%，二氧化硫、化学需氧量、氨氮、氮氧化物完成省下任务，烟尘、粉尘排放量均下降2%，万元工业增加值用水量下降5.2％。以上指标，约束性指标是刚性的，必须保证完成。部分预期指标的增幅比往年有所下调，主要考虑到宏观经济下行的大背景和我市连续多年高增长后需要有一个平稳调节阶段的实际。我们要更加自觉地把握和贯彻市委“打基础、利长远、惠民生”的总体要求，努力做到当前和长远相兼顾，经济和社会发展相协调，总量做大和民生改善相同步，真正实现科学发展。

实现以上目标，我们要重点抓好以下工作：

一、继续大上项目，保持经济持续稳增长实践证明，经济不发展，什么事也办不成。我市仍是欠发达地区，仍是全省最大的贫困地区，保增长、促发展是政府工作的根本任务。我们要毫不动摇地坚持上大项目、上好项目、上新项目，全面落实“项目推进年”各项要求，实行项目储备、签约、落地、开工、投资、投产“六位一体”整体推进，今年的目标是：项目储备完成10000亿元，项目签约、项目落地、项目开工、项目投资、项目投产分别完成1500亿元。今年共筛选确定市级重点项目173个，这些项目是稳增长的基础、调结构的抓手、惠民生的支撑，必须紧紧扭住不放，务必开工建设。

1、传统产业要升级改造，继续做大做强。传统产业既是吕梁市域经济的核心支撑，也是我们继续发展的坚实基础。坚持扩张规模和改造提升双管齐下，从而使我们的优势更优、强项更强。今年和今后一段时间要重点向“五大”推进。一是大煤焦。抓紧大矿建设，今年要重点加快临县晋煤太钢600万吨、临县美锦600万吨、交城西山500万吨、中阳汾西中泰400万吨、石楼中煤300万吨、文水潞安王家庄180万吨等大矿开工建设，改造扩建柳林华晋焦煤800万吨煤矿，完成全市22个矿井续建任务，使我市第二梯次的大矿尽快建成。抓好煤炭洗选，今年要建成临县霍电1000万吨洗煤、兴县华电1000万吨洗煤、柳林华晋800万吨洗煤、临县晋煤太钢600万吨以及交口麟达、易成泰、晟凯1500万吨洗煤项目，真正做到原煤不出市或少出市。抓住焦炭整合，加快孝义新型煤化工园区建设，推进中阳福裕、交城华鑫、文水海威和金地以及石楼中煤等焦化项目的进度，同时兼并整合中小焦化厂，把我市49户焦化企业整合为25户，使我市焦炭产业不仅能在低谷中挺住，还要能得到进一步的发展。二是大电厂。发电既是转化，也是转型。我们要全面打响吕梁电厂建设这一历史性战役，开工一批，上报一批，准备一批，真正迈出“输煤”变“输电”坚实的一步。年内要建成已由国家核准的交城国锦煤矸石电厂；加快已取得路条的汾阳国峰、文水国金、临县京能三个电厂的建设进度；争取已上报国家发改委的中阳大唐桃园、兴县国电、方山联盛、交口华瑞、孝义新阳、离石中河等6个低热质发电项目的开工；进一步做好柳林森泽和鑫飞、交城中电美锦、临县中电新民、兴县中铝华润、离石中电、中阳信发等电厂项目的前期工作。使全市电力装机容量尽快达到1000万千瓦，真正由挖煤大市向发电大市转变，并为滞销的煤炭开辟一条量大而稳定的销路。三是大铝铁。铝系方面，今年建成投产兴县中铝、孝义信发两个氧化铝项目，推进孝义信发、交口信发、中阳信发、兴县华润中铝新上4个铝项目，开工建设交口道尔低品位铝土矿综合利用和中铝铝基新材料、方山铝矾土深加工及铝制新材料等项目，使全市铝系产业得到大力发展。钢铁方面，总投资140亿元的孝义楼东焦钢一体项目已于最近全面开工建设。交城华鑫矿焦钢铸一体项目、美锦特种钢和铁合金项目也要开工建设。这样，铝铁很快会成为我市继煤炭之后的第二大支柱产业。四是大酒业。“汾酒城”要全面投产，使汾酒新增产量10万吨。下大力气整合文水散酒和原酒生产，启动优质白酒原酒集聚区建设，统一品牌，统一销售，形成我市又一10万吨级的酒业“龙头”。五是大园区。产业要发展，必须进园区。要努力把孝义梧桐、交城夏家营、文水百金堡三个省级园区和“一县一园”的工业园区做大做好，发挥集约、规模、服务、管理的作用。要重点突破省委、省政府力推的兴县华润中铝循环经济园，年内争取开工总投资约450亿元的1000万吨煤矿、204万千瓦电厂、200万吨氧化铝、100万吨电解铝、100万吨铝材、400万吨水泥等一揽子项目，使之成为全省第一大循环经济园区。

2、新兴产业要大力发展，推动循环转型。要把推动高碳资源低碳发展，黑色煤炭绿色发展，大力培育壮大新兴产业，作为全市经济发展的主引擎。一是延伸煤化工。以孝义鹏飞、金达，交城美锦、中阳福裕、离石大土河等企业为载体，建成一批“煤化工示范企业”，形成从源头到终端的相互衔接、不断延伸的“全产业链”，实现真正意义的转型。二是做大制造业。开工建设交口安华百万吨铸造加工项目，加快孝义华夏电动农用车电机及锂离子动力电池和金晖矿山煤机制造项目，争取岚县三鑫汽车配件总装生产线建成投产，开工建设文水华一重工大型铸造件项目，新上交城利虎汽车安全玻璃和太阳能聚光热发电用反射镜项目，促进我市装备制造业发展壮大。三是开发新能源。继续加快临县年产5亿立方煤层气项目和气化湫川项目,开工建设石楼20亿立方天然气项目，争取上马汾阳中节太阳能光伏电站和文水、岚县、方山风电项目，使我市太阳能和风力发电实现“零”的突破。四是发展新材料。进一步加快离石环保纸项目建设步伐，开工建设汾阳广东长城工艺陶瓷、岚县生物质材料加工、离石特种电缆等项目，实现我市新材料产业重大突破。五是壮大服务业，开工吕梁新城物流组团建设，加快汾阳阳城商贸物流经济开发区、孝义孝龙煤炭综合物流园区建设步伐，开工建设汾阳香港汉业汽车商务广场、孝义义乌商品交易国际博览城和华美新天地商业广场、交口中心商贸区、离石“居然之家”和天源物流等项目，促进我市第三产业繁荣发展。六是提升旅游业，重点抓好庞泉沟、碛口、北武当、苍儿会旅游综合开发，做好山水自然游、黄河风情游文章。同时做好汾酒城、杏花村镇的酒文化和旅游的开发，策划“印象汾酒”实景剧，打造我市特色旅游品牌。

3、高科项目要重点突破，引领结构调整。加快提高全市创新驱动能力，把发展高新技术产业摆在转型发展、调整结构的核心位置，加快引进一批科技含量高、资源消耗少、驱动能力强、带动能力大的高新技术项目，加快培育一批高科技企业。今年重点要进一步深化我市与国防科技大学的战略合作，全面推进军民融合协同创新，以研究院为平台，年内引进两院院士1-3名、知名专家10名以上，建立院士工作站、博士后流动站等科技研发机构，搭建无人矿山试验平台，建设能源互联网、云计算中心示范基地。切实抓好柳林李家湾光电子高新技术园、离石无人飞机研发生产、临县高性能计算机电子产业园等高新技术项目建设，力争在不远的将来，吕梁高新技术产业得到突飞猛进，为我市经济转型做出巨大贡献。

4、大上项目要攻坚克难，坚决扭住不放。各级领导要带头抓项目，把足够的精力放在项目建设上。重点要在审批、开工、进度、竣工、投产等关键环节上下功夫，特别是要在立项、环评、土地“三大关”上扭住不放，攻坚克难，协调解决各种问题，确保项目建设顺利推进。

二、倾力支持“三农”，走出产业扶贫新路子到现在，我市还有88万年收入在2300元以下的贫困人口，即使不在贫困线以下，多数农民的日子过得也并不宽裕。因此，做好“三农”工作是我们全部工作的重中之重，确保农民增收又是“三农”工作的核心。

1、不断创新生产经营体制。认真贯彻今年中央1号文件精神，一是积极培育专业大户。新增补贴要重点向新型生产经营主体倾斜，支持和鼓励承包土地向专业大户、家庭农场、农民合作社流转，发展多种形式的适度规模经营，解决农业经营规模小、生产效益低的问题。二是切实完善经营体系。大力支持发展多种形式的新型农民合作组织，支持农民合作社、专业服务公司、专业技术组织、农民经纪人等为农业生产经营提供低成本、便利化、全方位服务，促进农业生产集约化、专业化、组织化、社会化，破解农户分散生产与大市场有效对接的难题。三是不断健全保障体制。加强农业技术推广、动植物疫病防控、农产品质量监督等能力建设，完善以工扶农、以企帮村办法，完善农村金融服务，增加农业农村资金、技术、人才等生产要素的流入。

2、切实加大“三农”资金投入。公共财政要切实保障农业、倾斜农民。今年市本级预算安排2.4亿元，比去年进一步增加，重点用于农业农村基础设施建设、农业产业化发展、扶贫攻坚、农民增收等关键领域。各县（市、区）也要把支农支出列入预算，并尽可能多拿多投，以强大的资金投入作保障，促进农业增效、农村进步、农民增收。

3、精心培育特色主导产业。扶贫首在“扶业”，致富必须产业先行。在抓好“一村一品”、“一县一业”的基础上，重点启动实施“8+2”产业化增收工程。“8”就是培育壮大核桃、红枣、杂粮、蔬菜、马铃薯、食用菌、中草药和畜牧等8大产业，“2”就是抓好市场开拓和农产品深加工两个突破口。一是建设标准化生产基地。在优势产区划定专门区域，实行标准化生产，提高品质，扩大产量，形成稳定的商品规模。特别是食用菌、中药材等新产业要实现突破。食用菌要利用农村大量空闲的窑洞，由汾阳山宝食用菌公司兜底包销，发动农民积极栽培，增加有效收入。今年先在临县、石楼、离石、汾阳、中阳、方山、交口7个县（市、区）利用5000孔废弃窑洞、共计发展15万平方米的蘑菇生产规模，实现既利用废弃窑洞、又增加农民收入“一举两得”的效果。中药材要作为发展林下经济的主打产品，依托山西振东药业集团，实行完全的订单生产，今年至少发展1万亩；设施蔬菜要在巩固去年成果的基础上，今年以平川四县（市）为主体，新发展2.5万亩，形成面向省城、服务吕梁的“菜篮子”基地。核桃仍要扩面积，红枣仍要上烘干，养鸡、养牛、养猪仍要重增量，养羊要有大突破。二是打响有机绿色品牌。重点在提高农产品质量上下功夫，注册商标，组织有机、绿色产地和产品认证，推动我市特色农产品进入高档序列，占领高端市场，大幅度提高附加值。三是畅通销售渠道。创新营销模式，建立销售平台，加强产品包装和宣传，提高我市农产品知名度和市场影响力。为此，要精心组织，办好汾阳第七届世界核桃大会；发挥主产地优势，开工建设汾阳核桃批发市场，临县、离石、柳林红枣批发市场，岚县土豆批发市场、孝义农副产品批发市场、离石农产品仓储物流交易市场。同时，要继续壮大农业产业化龙头企业，以公司带农户，以产业促增收。

4、大胆闯出扶贫开发新路。一要抓机遇。紧紧抓住国家把吕梁山列为全国11个集中连片特困区的机遇，积极对接国家扶贫政策，以兴县、岚县、临县、石楼、方山为扶贫攻坚主战场，制订规划，明确重点，承接产业，放大效应。二要闯新路。按照省委提出的企业帮扶农村的战略思路，积极鼓励和支持大企业参加产业扶贫活动，以乡镇为单元，以土地流转为前提，加快促进农业产业化、农村公司化、农民工人化，大幅提升农民经营性收入、资产性收入和工资性收入。认真总结我市已有的好做法，积累经验，逐乡推开，覆盖全市，真正闯出一条扶贫攻坚的新路子。同时，要继续做好移民搬迁、整村推进和劳务输出等扶贫工作，通过综合实施以上措施，确保全市贫困农民的人均纯收入增幅高于全市农民人均纯收入的增幅，全市农民人均纯收入的增幅高于城镇居民人均可支配收入的增幅，力争今年再减少低收入贫困人口13万人。

5、全面推进新农村建设。按照“生产发展、生活宽裕、乡风文明、村容整洁、管理民主”的总要求，继续大力推进新农村建设。今年要完成集中连片示范区11个、打造高标准新农村100个、新农村重点推进村350个。

6、均衡补短促进协调发展。区域不均衡的问题目前在我市仍很突出，既有全省最好的县，也有最差的县，其中石楼的财政收入仍排在全省倒数第三位。所以，要把扶持石楼、补起短板、产业突破作为重要工作来抓。今年在石楼开工建设总投资78亿元的天然气项目，中煤总投资160亿元的煤焦电材一体化项目，要积极引进多家大企业到石楼去投资上项目。同时，市财政出资，帮助石楼县开展煤田勘探，掌握资源底数，促进招商引资，以产业发展真正带动石楼的脱贫脱困，逐步缩小区域发展差距，使之跟上全市快速发展的步子。

各位代表，农业是本，农民如天。我们一定要满怀真情，满腔热情，尽心竭力，真心实意，尽可能把更多的钱花在“三农”上，把最大的功用在“三农”上，努力把“三农”的事情办得更好，真正让农业的地位强起来，让农村的面貌美起来，让农民的日子好起来！

三、扩城增容提质，抢抓机遇推进城镇化习近平总书记在中央经济工作会议上说，“城镇化是我国现代化建设的历史任务，也是扩大内需的最大潜力所在”。李克强总理最近说，“未来几十年，中国最大的发展潜力在城镇化。”目前，我市城镇化率仅为41.5%，分别比全国、全省低10.9个和9.5个百分点。城镇化严重滞后既是吕梁长期贫穷落后的现实表现，又是加速现代化进程的最大障碍。当前，城镇化的机遇又一次来临，我们一定要有紧迫感和责任感，一定要有时不再来的机遇意识、奋力赶超的坚定信念，抢抓机遇，以建设吕梁新城为龙头，加速全市城镇化进程。

1、抓紧推进大县城建设。各县（市、区）都要抢抓机遇，以建设“大县城”为目标，全面推进规划转型、新区示范、旧区提质、城乡清洁、宜居创建等工程。积极开展卫生县城、园林县城等品牌创建活动。持续整治“脏乱差”，不断提高城镇管理水平。城镇化以城市群为主体形态，要推进“离柳中方”、“汾孝”城镇组群发展，支持一些经济强镇、区域重镇、文化名镇加快发展，使全市城镇化率提高1.7个百分点。在小城镇建设上，今年要重点与汾酒厂合作，抓好汾阳杏花村古镇的规划建设，形成名酒与古镇、名酒与旅游的互动，打造一座酒香千年、杏花百里、底蕴深厚的文化名镇。

2、加快吕梁新城建设。一是规划设计先行。由上海同济大学用一年的时间进行了整体规划，并聘请国内外知名的设计单位进行了组团和单体设计，在离石北川河大桥以北、方山峪口镇以南，规划出约30平方公里共11个组团，从南到北依次是南部综合组团、火车站综合组团、学院教育组团、南部生活组团、金融商业组团、行政中心组团、北部生活组团、总部基地组团、物流商贸组团、休闲娱乐组团和军事组团。二是重点建设项目。从今年开始要陆续开工建设五项“十大公益工程”，分别是：十大市政工程，即行政中心、文化博览中心、会议中心、政务便民中心、体育中心、吕梁广场、吕梁火车站扩建及广场、大武火车站及广场、汽车站、东西山防洪工程。十大教育工程，即科技职业技术学院、高等师范专科学校、技师学院、特教学校（聋哑、残疾）、学院区综合图书文体馆、1所高中、5所初中、10所小学、20所幼儿园、市委党校；十大公园工程，即东山森林公园、西山森林公园、北川河景观公园、七大湖面公园、三大湿地公园、休闲娱乐公园、滑雪冬运公园、儿童乐园、采摘园、吕梁英雄军事主题公园；十大交通道路工程，即3纵、20横、15座桥、100条街巷、209国道改线、大武到交城高速、有轨电车、十大停车场、环城高速新城连接线、飞机场新城连接线；十大医院、福利院工程，即大医院、中医院、儿童医院、妇幼保健院、疾控中心、120急救中心、中心血站、荣军医院、综合福利院、敬老院。今年首先开工建设包括学校、医院、道路、车站、文体设施、水电暖气、污水垃圾处理、通讯管网、防洪清淤、安置房等30多项重点工程，基本搭起新城框架。三是保障群众权益。严格依法办事，抓好政策落实，统筹做好补偿兑现、户口转换、低保救助、就业培训等一系列关键环节，特别是要先行建设安置房，保证群众按时回迁；建好商铺房，保证被拆迁户每户分得10平米经营房；优先安排失地适龄农民在新城就业，环卫、保安、物业、绿化等岗位一律留给当地群众，使他们利益不受损、生活有保障、生产能发展，为新城建设顺利推进打好基础。四是创新筹资模式。要采取多种形式的筹资融资和建设方式，特别是要鼓励民营资本和社会资金进入新城建设。五是加强质量管理。加强工程质量和工程廉政监督管理，利用我市新建的公共资源交易中心和工程廉政监管中心联合平台，加强对新城建设工程招投标、土地交易、政府采购等环节的监管，确保新城建设优质高效廉洁，真正经得起历史的检验。六是坚持“两个同步”。即同步规划改造老城区和同步规划产业发展布局。在建设新城的同时，要抓紧修订完善离石老城区规划建设，同步启动旧城改造，做到新老城区功能互补、共同发展。同时，要同步规划城区的产业布局，以产业来支撑未来城市的发展。

3、健全城镇化保障体系。城镇化首先是农民市民化，更重要的是基本公共服务均等化，要实现就业方式、社会保障等一系列由“乡”到“城”的转变。我们要研究出台户籍制度改革实施办法，有序推进农业转移人口市民化，重点解决社会保障、子女上学、保障性住房等问题，真正让农民能进得来、留得住、过得好。

四、大搞基础建设，提供全面发展新保障基础设施是经济发展、社会进步、改善民生的重要支撑。要进一步加强基础设施建设，赢得发展先机，增强发展后劲。

1、建设立体交通。机场上，吕梁机场今年上半年实现校飞，下半年正式开通北京、上海、成都等地的航线。铁路上，中南大通道、太兴铁路、吕临铁路2014年建成。今年重点加快27个战略装车点及专用线的建设，扩建吕梁火车站，争取开工建设交口到孝义的铁路，力争年内开通吕梁到太原的城际动车。公路上，西纵、环城两条高速和太佳高速黄河大桥10月份正式通车；今年重点争取开工建设吕梁新城至交城、西纵离石至交口、交口至汾阳、兴县蔡家崖至黄河的高速公路；进一步做好兴县黑峪口至静乐、石楼到交口高速公路的前期工作；开工太佳高速临县、方山、岚县（2条）共四条连接线一级路建设；开工并完成兴县蔡家崖到魏家滩一级路建设；完成209国道吕梁新城过境、307国道柳林城区段改线、340省道中阳城区段改线等公路改扩建工程；开工建设新城汽车站。加强沿黄公路、县乡公路、乡村公路改造养护，确保公路交通便捷畅通。

2、加强水利建设。继续实施“千井富民工程”，今年再新打200眼灌溉深井。完成龙门供水等骨干工程年度任务，继续推进中部引黄和沿黄提灌工程，争取方山峪口沟、交城魏家沟、兴县阳湾则、柳林高红等4座水库立项建设。新建人畜饮水安全工程268处。还要全力争取列为全国人代会议案的碛口水利枢纽工程。

3、完善电网布局。加快临县白文500千伏输变电工程，开工4座220千伏、9座110千伏输变电工程，扎实抓好农网、城网升级改造，进一步提高全市输变电能力。

4、加速“气化”进程。完成临县到离石、离石到太原、临县到保德煤层气输配建设并实现供气，并建成20个加气站。已开发的6个区块煤层气产能稳定达到15亿方。开工建设4个液化工厂，形成日加工200万方煤层气的能力，今年争取所有县（市、区）城区居民都用上天然气，加快“气化吕梁”步伐。

五、多办民生实事，促进人民生活上水平我市社会事业和公共领域历史欠账太多，与我们日益增长的经济实力极不相称，与人民群众改善生活的迫切愿望很不相符。因此，要大办、多办民生实事，大力发展社会事业，弥补差距，拉长“短板”，努力实现均衡发展，不断提高人民群众的生活水平。

1、加快发展社会事业。一是均衡发展各级各类教育。要建成去年已经开工的市直体育幼儿园，建成县城幼儿园20所、乡镇中心幼儿园50所，鼓励有条件的农村自办幼儿园。建成由市政府统贷统还建设的吕梁学院附中。力争交城、文水、兴县、岚县、临县、方山、石楼7个县通过省政府义务教育均衡发展验收，并完成薄弱学校改造工程。加快推进中专学校重组升级和高等教育建设发展，支持吕梁学院和汾阳医学院进一步做强做大，吕梁职业技术学院（孝义）下半年招生，开工建设吕梁高等师范专科学校、吕梁科技职业技术学院、吕梁高级技师学院和特殊教育学校，并配套建设学院区综合图书馆、文体馆。全面推进素质教育，着力提高教育质量。加强校园安全管理。扎实推进城市社区体育设施和乡镇体育健身广场建设，在新城建设体育场、体育馆、游泳馆，推动全民健身和竞技体育蓬勃发展。二是推进文化改革发展。切实加强思想道德建设，在全社会大力弘扬“吕梁精神”。巩固扩大文化体制改革成果，促进公益性文化健康发展。完善基层文化设施，实施文化惠民工程，积极开展送戏下乡活动。在新城今年开工建设大型文化综合体，包括展览馆、博物馆、图书馆、艺术馆、美术馆、科技馆、大剧院、青少年活动中心、老年活动中心、妇女活动中心、工人文化宫等文化场所。培育壮大文化产业，推动文艺创作繁荣发展。加强非物质文化遗产传承保护。三是大力发展医疗卫生事业。开工建设以吕梁大医院为主的医疗综合体。建成市人民医院门诊医技综合大楼、汾阳医院门诊大楼。推进乡镇卫生院和社区卫生服务机构标准化建设。继续深化医药卫生体制改革，全市60%以上的县级以上公立医院实施国家基本药物制度，新农合农民参合率保持在97%以上。加强地方重大疾病防治，提高全民健康水平。加强与北大医院等全国知名医疗卫生机构的合作交流，组织举办公益医疗活动。稳定低生育水平，提高出生人口素质。四是积极扩大就业。创造就业岗位，加强技能培训，帮助劳动者就地就近稳定就业。积极协调驻吕央企、国企、省企为当地群众提供就业岗位。今年城镇新增就业4万人，城镇登记失业率控制在4.2%以内。五是完善社会保障体系。提高城乡居民基础养老金，月领标准不低于65元；企业退休人员基本养老金提高10%。城镇居民医保和新农合财政补助标准提高40元、达到每人每年280元。城乡低保标准每人每月分别提高30元和24元，并做到应保尽保。提高用工最低工资标准。开工建设殡仪馆和公墓。新建各类保障性住房23094套，基本建成18442套。以吕梁新城建设为契机，同步建设机关工作人员住宅小区。要提高行政事业单位人员的工资水平，提高取暖补贴标准，提高公积金缴存比例，使我市这三方面均达到全省的高限。关心老龄和妇女儿童事业，支持工会、共青团、妇联等人民团体发挥更大作用，重视外事、侨务、港澳、对台工作，办好民族宗教事务，大力支持军分区、武警、消防、民兵预备役等军队和国防建设，加强军政军民团结和“双拥”工作，做好老干部工作，扎实抓好统计监测和第三次经济普查，统筹做好人防、防震减灾、档案史志、民政慈善等工作，充分调动各方积极因素，形成推动吕梁转型跨越发展的强大合力。

2、推进生态文明建设。我们要把生态文明理念和节能减排行动贯穿到工作的全过程和各个方面，突出解决好关系群众切身利益的大气、水、土壤污染等环境问题，努力在经济发展的基础上逐步改善生态环境。抓好造林绿化和生态治理恢复。今年要实施吕梁山生态脆弱区植被恢复造林、高速公路沿线绿化、新城东西山绿化和核桃林发展四项重点工程，全市完成造林68万亩，新发展核桃50万亩。继续坚持“一企一矿治理一山一沟”的做法，推进吕梁特色的荒山绿化、小流域治理等工程，积极开展采矿塌陷区治理，抓好北川河等重点流域综合整治，积极开展水生态系统保护与修复试点，严禁超采地下水。加强吕梁新城上游植被修复和水资源保护。推进节能降耗和减排治污。加大淘汰落后产能力度，引导重点企业实施技术改造，完成33万平方米建筑节能改造任务。打好打胜空气和水环境质量改善攻坚战，持续开展城市燃煤烟尘、扬尘整治和污染防治，重点加强 PM2.5 监测和预警工作。新建一批城市污水处理厂，县县建成生活垃圾处理场。积极防范重大环境污染事件，严厉查处环境违法行为。

3、办好“5+1”六件为民实事。去年，我市率先在全省启动和实施了“方便农民五件实事”，取得了显著成效，大大方便了农民的生活，得到了农民的一致好评。今年在加强管理、巩固成果的基础上，再新建农村幼儿园300所、洗澡理发室1000个、碾米豆腐房800个、安装太阳能路灯1000个村、红白理事厅1000个，力争三年全面完成并覆盖所有行政村。同时再增加一件实事，就是每个县（市、区）都要建成一所社会综合福利院，把鳏寡孤独、残疾智障人员和流浪儿童集中供养起来，彻底解决他们无依靠、无保障、流动分散的问题。这部分人员基本没有自我生存能力，是最为典型的弱势群体，对于他们的生活，政府不仅要管，而且必须兜底。这方面，市政府首先带头，已决定在新城投资2亿多元建一座全国一流的综合福利院，包括儿童福利院、社会福利院、荣军院、救助站等内容，床位总数达到1500支。各县（市、区）政府也要高度重视，今年要全部开工建设，尽快建成并正常运行。同时，要抓好经费保障、日常管理工作，使现有敬老院、福利院正常运行。此外，我们要扎实办好省政府“五件实事”中与我市“六件实事”不重合的另外三件实事，即年内完成农村困难家庭危房改造8850户、特困群众易地搬迁2万人、乡村清洁工程1300个村，真正把实事办实、好事办好，惠及广大人民群众。

各位代表，民生所指，民心所向。为民造福是政府全部工作的最终目的，也是检验政府工作成效的根本标准，我们就是要这样从关系群众切身利益的实事入手，一件一件抓落实，坚持不懈抓下去，聚沙成塔，久久为功，让发展成果惠及更多的人民群众！

六、推进改革开放，不断增强发展新活力

1、转型综改。紧紧围绕产业转型、生态修复、城乡统筹、民生改善“四大任务”，突出“走出转型路径、改革体制机制、强化组织保障”三个重点，扎实推进综改试验。加快完成省确定我市为“市域城镇化”板块的实施方案编制及报批工作，着力抓好年度行动计划的组织实施，扎实抓好孝义、兴县2个省级试点县，中阳、岚县2个市级试点县以及“一市两园”、“一县一企”等试点工作，大力推进试点园区以及标杆项目建设。大胆创新产业园区化集聚、项目园区式审批、环境容量市场化配置、土地占补平衡、地方金融体系培育和扶贫模式创新等方面的体制机制，努力把我市建成重度资源型地区产业升级示范区、生态脆弱地区生态保护修复样板区、集中连片贫困地区全面小康地区。

2、机构改革。根据今年国务院机构改革的原则和要求，按照统一部署，积极推进市、县（市、区）两级政府机构改革和职能转变。要简政放权，减少审批事项，减少资质资格许可，减少行政事业性收费，放宽工商登记。按照政府职能向创造良好发展环境、提供优质公共服务、维护社会公平正义转变的要求，适应加强市场监管、提供基本社会保障的需要，着力解决政府与市场、政府与社会的关系问题，着力解决职能交叉、推诿扯皮、管得过细的问题，真正做到该管的管住管好、不该管的不管不干预，切实提高政府管理科学化水平。

3、招商引资。要拓展视野找项目，扩大市外境外客商在吕梁的投资规模；放大优势孵项目，以优势资源换项目、换投资、换技术、换人才；吃透政策育项目，围绕国家重点扶持鼓励领域，积极挖掘和培植项目；用好平台引项目，既要用好市内平台，也要在市外平台上做文章，打造具有吕梁特色的招商引资“磁力场”。以企业为主体、以园区为平台，推动引资、引技、引智有机结合，提高招商引资的综合优势和总体效益。市、县两级都要适时组织安排一次较大规模的招商活动，提高针对性和签约率，加快引进一批科技含量高、资源消耗少、税赋贡献大、带动能力强的大项目、好项目、新项目。进一步加大招才引智力度，引进高新技术人才、管理人才以及我市急需的各类实用人才。扎实抓好外贸平台建设，调整产品进出口结构。全面落实与太原海关的战略合作协议，提高通关便捷程度，促进外向型经济健康发展。

4、给力民营。民营经济是吕梁经济的活力所在。我们要继续加大支持力度，给环境、给支持、给优惠、给信心、给信任，保证各种所有制经济依法平等使用生产要素、公平参与市场竞争、同等受到法律保护，着力破解民营经济获批资源、征占土地、贷款融资、引进人才等方面的难题，积极扶持和引导民营企业家投资实体经济、承接转型项目、吸纳高新技术，促进我市民营经济加快转型升级，实现二次腾飞。

5、创优环境。着力营造有利于发展的法制环境、市场环境和社会环境。进一步解放思想、更新观念，在全社会形成开放包容、合作共赢、支持创业、鼓励创新的良好氛围，向一切投资创业者提供最大限度的支持和便利。推行“三个24小时”直通车制度，即重大项目、重点企业遇到重大问题时，24小时之内要报告分管副市长，特别重大的要报告市长;接到报告后由分管副市长牵头研究，24小时内提出解决问题的建议和方案;再在24小时内对相关企业所报事项予以答复反馈，以优良的环境引来投资、留住客商。

七、落实监管责任，确保安全稳定上台阶

1、全面加强安全生产。安全是一切工作的底线，也是最大的民生工程，只有起点，没有终点。严格落实责任。常怀敬畏生命、敬畏责任、敬畏制度之心，进一步强化政府的监管责任，严格落实企业的主体责任，把安全责任逐级分解到每个岗位、每个环节，做到事事都有责任制、人人都有责任心。深入整治隐患。突出重点领域、重点行业、重点部位、重点时段，继续深入开展专项整治，深化隐患排查治理，重拳出击，重典治乱，深入开展“打非治违”，严厉打击私挖滥采行为，进一步规范生产、建设和经营活动，促进安全隐患排查治理制度化、规范化、常态化。强化应急救援。全面加强应急救援基础建设和能力建设，着力提高应对突发事件的科学化水平。严格责任追究。严格落实安全生产“一票否决”制，坚持监管前移、问责前移，严肃查处各类安全事故，依法追究相关责任人的责任，促进全市安全生产形势持续稳定好转。

2、全力维护社会稳定。巩固扩大“三大活动”成果，建立健全群众诉求表达、利益协调、权益保障机制。深入开展基层组织纪律作风集中整治年活动。完善流动人口、特殊人群、非公经济组织和新社会组织以及信息网络的服务与管理。强化食品药品安全监管，确保人民群众饮食安全、用药安全。完善治安防控体系，始终保持对重大刑事犯罪的严打态势，全力维护政治稳定、社会安定。

八、大力改进作风，打造人民满意的政府

1、坚持依法行政。认真执行人大及其常委会的决议决定，自觉接受其法律和工作监督，支持人民政协履行政治协商、民主监督、参政议政职能，认真办理、落实人大代表的议案、建议和政协委员的提案，主动听取和采纳民主党派、工商联、无党派人士的意见建议，支持人民法院、检察院的工作。深入推进“六五”普法教育，形成浓厚法制环境。主动接受社会公众和媒体监督，让权力在“阳光”下运行。

2、提高行政效能。政府工作要做到抓铁有痕、言出必行、说到做到、落实高效。凡是作出的决定要严格执行，凡是部署的工作要立即完成，凡是承诺的事项要全面兑现，凡是确立的目标要圆满实现，自觉养成马上办、高效办、成功办的政风，坚决克服不想干、不会干、不敢干的惰性，以强有力的执行力、公信力、影响力确立政府的崭新形象。认真落实首问负责制、限时办结制、服务承诺制、责任追究制，改进绩效考核，健全激励机制，促进政府各部门规范高效运转。

3、坚持廉洁从政。健全完善重大问题决策程序和规则，提高政府决策科学化、民主化、法治化水平。认真落实党风廉政建设责任制和领导干部廉洁自律各项规定，扎实推进“一网八平台”惩防体系建设，严厉查处各类违法违纪案件，坚决纠正损害群众利益的不正之风。坚持厉行节约，反对铺张浪费，严格预算管理，压缩“三公”开支，把有限的资金尽量用到民生上去。

4、改进工作作风。严格执行中央关于改进工作作风、密切联系群众的“八项规定”，以良好的政风促行风、带民风。市政府领导要做好“五带头”：一是带头下基层，每人每年下基层搞调研不少于60天，调研活动要注重实效，真正发现问题、解决问题；二是带头包项目，每人联系一项重点项目，包审批、包开工、包进度、包投产，一包到底，不达目的不松手；三是带头抓扶贫，年内要为所住贫困村引进一户企业，发展一项产业，促进农民增收；四是带头解难题，在各自分管领域认真解决事关民生和发展的突出问题，或解决影响全局的复杂问题；五是带头办实事，每人领办一项，牵头负责，抓好落实，按时兑现。通过领导干部以身作则、率先垂范，带动政府系统工作作风进一步好转。

各位代表，新的目标催人奋进，新的征程任重道远。让我们在中共吕梁市委的坚强领导下，坚持“打基础、利长远、惠民生”的总体要求，解放思想，改革创新，齐心协力，锐意进取，奋力推进吕梁转型跨越发展，为加快实现稳定脱贫、全面建成小康社会的宏伟目标而努力奋斗！
